# The misuse of codeine containing medicines: Perceptions and behaviours of qualified pharmacy professionals

**DOI:** 10.4102/safp.v66i1.5862

**Published:** 2024-05-10

**Authors:** Elmien Bronkhorst, Munira Adamjee, Madan Poka

**Affiliations:** 1 Department of Clinical Pharmacy, School of Pharmacy, Sefako Makgatho Health Sciences University, Pretoria, South Africa

**Keywords:** codeine, pharmacy personnel, misuse, dependence, behaviours

## Abstract

**Background:**

Pharmacy professionals working in community pharmacies frequently provide pharmacist-initiated therapy, including codeine-containing medicines. Codeine is an opioid with great potential for misuse, adding to the global opioid epidemic burden. Professional pharmacy personnel are the first intervention point in relation to management of codeine use. This study highlights the importance of pharmacy professionals’ perceptions and behaviours in combatting the opioid epidemic.

**Methods:**

A descriptive cross-sectional study was conducted. Simple random sampling included pharmacy professionals in the metropolitan city of Johannesburg. An electronic questionnaire was distributed via e-mail and data analysed descriptively.

**Results:**

Findings indicate that pharmacy personnel routinely ask patients about codeine use (*n* = 48; 53.9%), avoid dispensing over-the-counter (OTC) codeine as an initial treatment (*n* = 61; 69%) and express confidence to identify and manage codeine misuse (*n* = 69; 77.5%). Despite this, increased patient demands for OTC codeine (*n* = 69; 77.5%) were concerning, highlighting the ease of availability from internet sources (*n* = 76; 85.4%) and multiple pharmacies (*n* = 84; 94.4%). Apprehension about the lack of patient awareness on adverse health consequences (*n* = 66; 74.2%) and the risk of codeine dependence (*n* = 79; 88.8%) was expressed.

**Conclusion:**

Growing concern regarding availability and accessibility of codeine-containing medicines within the community pharmacy sector is highlighted. Adverse health consequences of codeine misuse and dependence are not understood by customers and the ineffective information provided by pharmacy personnel was highlighted as a concern.

**Contribution:**

The results of this study give insight to the influence of dispensing personnel’s attitude towards the growing challenges with respect to codeine containing medication abuse.

## Introduction

Community pharmacies in South Africa (SA) can be either independent or form part of pharmacy chains. They are predominantly located in urban areas, where they serve the private healthcare sector, where many patients have medical insurance. However, pharmacists, as well as qualified post-basic pharmacist assistants (QPBPA) (under the supervision of a pharmacist), working in this environment, frequently provide pharmacist-initiated therapy and sell over-the-counter (OTC) medicine to the insured and uninsured patients.^1^ This often includes pain medicine containing codeine. As per SA regulations, codeine is scheduled as S2 medicine, which should be stored behind a counter in a pharmacy and may only be supplied by a pharmacist or QPBPA under the supervision of a pharmacist.

Codeine is an opioid commonly used in the treatment of mild to moderately severe acute and chronic pain, dry cough and diarrhoea.^2^ The ease of availability, largely because of the reduced regulations of codeine in most countries, including SA, has greatly increased its potential for abuse and misuse.^3^ Codeine-containing combination medicines can be a relatively safe form of treatment.^4^ However, the lack of public awareness of its habit-forming potential^5^ and the ease of access to OTC medicine have resulted in the perception that codeine-containing products are harmless.^6^ However, excessive or long-term use of these products risks the development of tolerance with related health harms like gastro-intestinal ulcers and haemorrhage. Immediate side effects of codeine include constipation and drowsiness, which poses risk when driving and performing daily tasks.^4,5^ Furthermore, it poses a major public health, social and individual problem. It can contribute to deliberately or otherwise harmful effects in physical, mental, emotional or social impairment, which can be considered an aggravating factor for economic crises.

According to the World Health Organization (WHO) pain ladder, the treatment of mild pain includes paracetamol and non-steroidal anti-inflammatory agents (NSAIDs) and does not include opioids. Weak opioids like codeine, dihydrocodeine and tramadol, with or without paracetamol and/or NSAIDs, can be used to manage moderate pain for a short duration.^7^ Despite the WHO pain management guidelines, there is an increased tendency to use codeine-containing pain medication as first-line treatment.^8^ In many parts of the world, substantial numbers of patients are purchasing and consuming codeine in OTC products. Increased public health measures are required to improve the safe use of such products. There are no known validated screening tools for identifying pharmacy customers who are at risk of codeine misuse. Efforts to tackle the misuse of codeine may require serious consideration of up-scheduling and strict adherence to the regulations pertaining to restricted supply,^9^ advertising of codeine-containing products and providing information upon dispensing these products to communities.^5^ Additional regulatory measures such as record keeping and direct pharmacist intervention at point of sale may contribute positively to public deterrence of codeine misuse.^3^

Pharmacists play an important role in pain management by providing such services as medication reconciliation, drug monitoring and assessment, patient and healthcare provider education and counselling.^10^ Various studies highlight pharmacists’ opinions and experiences when dealing with the dispensing of codeine products and the difficulties experienced in attempting to improve patient awareness and knowledge of codeine misuse and dependence.^11,12^ Limitations in communication between pharmacists and customers have been highlighted as a serious concern.^11^ Pharmacists describe pharmacy hopping (using different pharmacies to obtain OTC medication) and difficulty in the relationships between the pharmacist and prescriber, as barriers to identifying customers potentially misusing codeine.^3^ Limited literature on the strategies followed by pharmacists to improve the management of codeine misuse and dependence in SA is available. Therefore, this study aims to examine the perceptions and behaviours of qualified pharmacy professionals (pharmacists and QPBPA) on the misuse of codeine-containing medicines in community pharmacies of Johannesburg, South Africa.

## Methods

### Study design

This study employed a descriptive cross-sectional quantitative design with simple random sampling.

### Study site

According to Nyamuzihwa et al.,^13^ there are a total of 1264 community pharmacies across Gauteng province. The study was conducted within community pharmacies of Johannesburg metropolitan municipality, Gauteng province, South Africa. The Gauteng province consists of 5 metropolitan municipalities of which Johannesburg metropolitan municipality is the biggest, representing a population of 4.4 million in 2018.^14^

### Study population

The target population included two professional groups, pharmacists and QPBPAs, who were actively involved in dispensing OTC medicines and counselling clients in the selected community pharmacies. Data obtained from the South African Pharmacy Council (SAPC) indicated the total population size of the above professional groups to be 2566 in the Johannesburg metropolitan municipality.

### Sample size and selection

The sample size calculated using Raosoft^®^ was a total of 335 for both the professional groups, pharmacists and QPBPAs, with a confidence interval of 95% and a 5% margin of error. Simple random sampling was conducted by identifying all pharmacists and QPBPAs within the Johannesburg community pharmacy sector. The names were filtered and selected from the SAPC list of email addresses of the two categories of registered pharmacy staff working within the community pharmacies in the Johannesburg metropolitan municipality.

### Study period

The study was conducted over 5 months within the community pharmacy sector of Johannesburg metropolitan municipality.

### Data collection instrument

The data collection instrument used in the study was a Google Forms™ questionnaire consisting of participant demographical data and a section on the perceptions, behaviours, practices and counselling methods on the misuse of codeine-containing medicines. The questionnaire was derived from a previous study by Foley et al.^15^ A Likert-scale was used to establish pharmacists’ and QPBPAs’ perceptions, behaviours and practices. The online questionnaire was administered electronically via email, using Google Forms™ and was analysed descriptively. The data collection instrument was validated by means of a pilot study conducted among 10 professional group members working in the community pharmacy sector in a different city. Results of the pilot study were not included in the study results.

### Data analysis

Data were collected from the electronic platform Google forms™ and were exported into Microsoft Excel™ spreadsheets. Data obtained was analysed using SPSS (Statistical Package for Social Sciences) version 27, summarised descriptively to include means, standard deviation, frequencies and percentages. The results are presented in frequency tables and graphs. Analysis of variance (ANOVA) was also conducted to indicate any differences between the two professional groups, correlations and Chi-square to show any associations within the data. All statistical results were considered significant when *p* ≤ 0.05.

### Ethical considerations

Ethical clearance to conduct this study was obtained from the Sefako Makgatho University Research Ethics Committee (No. SMUREC/P/44/2022). Participant consent was received upon completion of the questionnaire. The identities of both pharmacists and QPBPAs participating in the study were kept confidential through the absence of personal details in the demographic section of the questionnaire. The collected data was stored securely and not used for any other purpose other than research purposes.

## Results

### Response rate

The questionnaires were emailed to 335 selected participants of professional groups (pharmacists and QPBPAs). A total of 89 responses (*n* = 89) were received, of which 69 are pharmacists (77.5%) and 19 QPBPAs (21.3%), giving an overall response rate of 26.6%. Two recipients (0.6%) opted not to participate in the study.

### Demographic data

The demographic information (Table 1) shows that the majority of the participants were female (*n* = 58; 65.2%) while the median age was 30–39 years. Most of the participants were Asian/Indian people (*n* = 36; 40.4%), and 32% were black South Africans. The median total number of years of experience was in the range of 10–19 years while the median for years of experience in a community pharmacy environment was 5–9 years. Half of all participants (*n* = 45; 50.6%) were employed full time within the community pharmacy environment, while 38 (*n* = 42.7%) practised as locums.

**TABLE 1:** Demographic data (*n* = 89).

Demographic information	Frequency (f)	%	Median	Range
**Profession (89 responses)**	-	-	-	-
Pharmacist	69	77.5	-	-
QPBPA	19	21.3	-	-
No response	1	1.1	-	-
**Age (years)**	-	-	30 to 39	20 to ≥ 60
**Gender (*n* = 89)**	-	-	-	-
Male	30	33.7	-	-
Female	58	65.2	-	-
Prefer not to say	1	1.1	-	-
**Ethnicity (*n* = 89)**	-	-	-	-
Black Africans people	32	36	-	-
Asian/Indian people	36	40.4	-	-
Mixed race people	2	2.2	-	-
Caucasian people	18	20.2	-	-
No response	1	1.1	-	-
**Overall years of pharmacy practice experience (years) (*n* = 89)**	-	-	10 to 19	0 to > 30
0–4	13	14.6	-	-
5–9	25	28.1	-	-
10–19	25	28.1	-	-
20–30	17	19.1	-	-
> 30	9	10.1	-	-
**Years of experience in community environment (years) (*n* = 89)**	-	-	5 to 9	0 to > 30
0–4	33	37.1	-	-
5–9	18	20.2	-	-
10–19	21	23.6	-	-
20–30	10	11.2	-	-
> 30	7	7.9	-	-
**Terms of employment (*n* = 89)**	-	-	-	-
Full time	45	50.6	-	-
Part time (contract)	6	6.7	-	-
Locum	38	42.7	-	-

QPBPA, qualified post-basic pharmacist assistant.

### Perceptions on codeine misuse and dependence

The majority of respondents (*n* = 72; 80.9%) are in agreement that patients believe that OTC codeine-containing medicines are safe. However, 78 respondents (87.6%) strongly agree that there is a potential for misuse of codeine-containing medicines. Many participants (*n* = 69; 77.5%) agree that patient requests for OTC codeine-containing medicines are increasing, while 63 (70.8%) participants showed concern about the ease of availability of OTC medicines containing codeine.

A significant number of participants (*n* = 84; 94.4%) recognised the potential for patients to buy codeine-containing medicines from multiple pharmacies, which may add to the burden of codeine misuse. A concern about the availability of codeine-containing medicines from the internet was shown by 76 (85.4%) participants. Sixty-eight (76.4%) participants disagree that doses of < 30 mg of codeine phosphate are not effective in the management of mild to moderate pain. Seventy (78.7%) participants agreed that OTC codeine-containing medicines have a greater potential for inappropriate use compared to prescribed medicines containing codeine, while 71 (79.8%) agree there is great potential for OTC codeine-containing medicines to be used as recreational drugs.

The problem of codeine misuse being as serious as that of stronger opioids is agreed upon by 79 (88.8%) respondents. There was no significant difference in responses on the development of codeine dependence being higher in females than males. More than half (*n* = 45; 50.6%) of respondents chose to remain neutral to this question.

**FIGURE 1:**
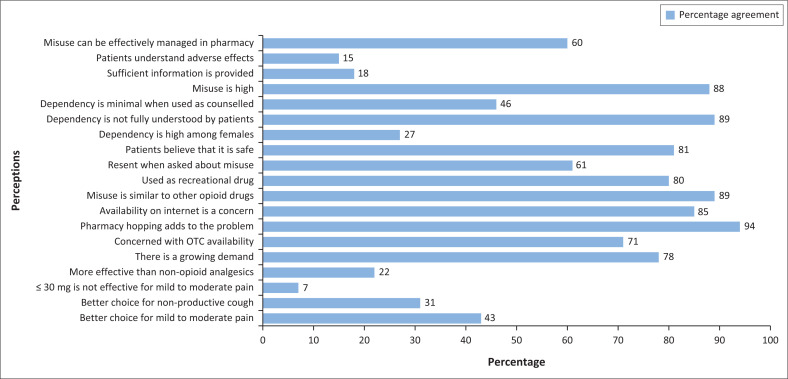
Perceptions of pharmacists and qualified post-basic pharmacist assistants on codeine misuse and dependence.

There is a great level of agreement that the risk of dependence is not fully understood by patients when taking OTC codeine-containing medicines (*n* = 79; 88.8%), while 41 (46%) participants agree that the risk of developing codeine dependence is low when taking codeine-containing OTC medication as counselled. However, 54 (60.7%) disagree that patients are given sufficient information on the use of OTC codeine-containing medication; 66 (74.2%) disagree that patients understand the adverse health consequences associated with continued and excessive use of combination codeine preparations. According to 53 (59.6%) participants, codeine misuse and dependence can be managed effectively within a pharmacy practice, while 24 (27%) disagree with this statement. Figure 1 illustrates statement items examining pharmacists’ and QPBPAs’ perceptions on codeine misuse and dependence as experienced in the community pharmacy environment.

Professional groups identified patient behaviours that triggered high suspicion of codeine misuse. Firstly, the patient behaviour that most frequently triggered a suspicion of codeine misuse was specific brand requests by patients (*n* = 37; 42%). Secondly, a history of misuse or addiction at 38.2% (*n* = 34) and pharmacy hopping was the third suspicion trigger for codeine misuse at 31.5% (*n* = 28).

### Behaviours and practices

**FIGURE 2:**
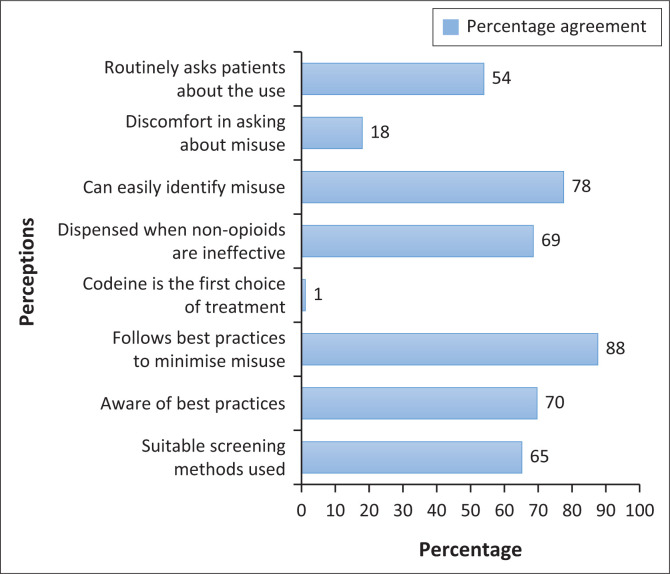
Behaviours and practices of pharmacists and qualified post-basic pharmacist assistants on codeine misuse and dependence.

Encouragingly, 61 (68.5%) of respondents feel comfortable asking patients about their codeine use, and 69 (77.5%) are confident that they can identify codeine misuse or dependence in their patients. Reinforcing this statement, 59 (66.3%) disagree on having difficulty in identifying potential problematic use of OTC codeine-containing medicines without the patient first informing them. Not dispensing OTC codeine-containing medicines to patients as an initial strategy was described by 64 (71.9%) respondents, while 61 (68.5%) respondents would dispense codeine-containing preparations following unsuccessful treatment with non-opioid analgesics. The number of participants who are fully aware of best practices in managing codeine misuse and dependence was 62 (69.7%) with 78 (87.6%) being willing to follow these best practices. The utilisation of suitable screening methods in order to identify inappropriate use of codeine-containing OTC preparations was practised by 58 (65.2%) respondents. Figure 2 illustrates statement items examining pharmacists’ and QPBPAs’ behaviours and practices on codeine misuse and dependence as experienced in the community pharmacy environment.

### Strategies in dealing with codeine misuse

**FIGURE 3:**
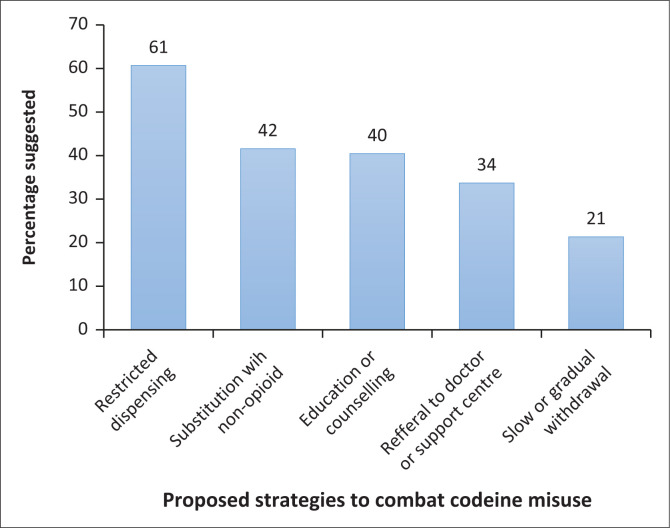
Strategies to combat codeine misuse.

The most frequently utilised strategy upon suspicion of codeine misuse, as confirmed by 54 (61%) respondents, is to restrict dispensing of the codeine product, by reducing the frequency of supply to a specific client. The second most practised strategy (*n* = 37; 41.5%) is to substitute with a non-opioid medication. Meanwhile, 36 (40.4%) participants would opt to provide education and counselling. Only 30 (22.5%) participants indicated referral to a doctor or support centre. The least practised being the (*n* = 19; 21.3%) option to withdraw the codeine-containing product slowly or gradually. Figure 3 is a graphical representation of counselling strategies practised by pharmacists and QPBPAs.

## Discussion

The study received 89 responses with an overall response rate of 26.6%. The response rate is in line with response rates from other web-based studies conducted among pharmacists in Malaysia, which showed response rates of 31% and 29.2%.^16^ In a study conducted on SA medical practitioners by Foley et al., 2018, a final response rate of 4.7% was achieved. Lower response rates have been documented for electronic studies (33.7%) as opposed to paper-based surveys (43.4%).^17^

Most of the participants were female and employed within the private community pharmacy sector. This is depictive of the SAPC 2018 gender distribution of female to male pharmacists in South Africa (62% female)^18^ and the SAPC 2015 data on area of practice illustrating that 68.3% of all pharmacists work within the community pharmacy private sector.^1^

Pharmacy professionals agree that the risk of dependence and adverse health consequences associated with excessive use of codeine preparations is not fully understood by patients. This is corroborated by Dada et al.,^19^ where the vast majority of patients do not view themselves as needing help or as experiencing addiction. Pharmacy professionals further indicated that this could be because of insufficient information being provided to patients. Most participants in this study have less than 4 years’ experience, which could have had an impact on the ability to provide sufficient information to patients (*p* = 0.006). According to Hoppe et al.^12^ scoping review, the years of experience is directly related to fewer perceived barriers to the provision of opioid interventions. Pharmacists with longer practice experience (3–10 years) were more likely to discuss misuse with patients as they may have a better understanding of the pharmacotherapy and management of chronic pain. Carney et al.^20^ highlighted the ineffectiveness of information provision models that currently exist for patients at the point of dispensing. The study further highlighted that the patients’ lack of interest in utilising current sources of information (patient information leaflets) is likely to reflect a general patient perception that these products are safe.

The study findings indicate that just over half of the pharmacy personnel routinely ask patients about their use of OTC codeine. Good dispensing practices are followed by most respondents, whereby they avoid dispensing OTC codeine products as an initial treatment. Most participants agree that they would only dispense codeine preparations following unsuccessful treatment with non-opioids. Despite this, 42.7% still agree that OTC codeine-containing medicines are a better choice for the treatment of mild to moderate pain. High levels of agreement were exhibited by the respondents showing great concerns about the increased patient demand for OTC codeine-containing products.

South African legislation published in the Government Gazette 40869, dated 26 May 2017, restricts dosage units of 10 mg codeine per tablet or capsule and 10 mg per 5 mL of codeine syrup,^9^ and the regulatory requirement dictates that sales be recorded in a S2 register.^3^ Despite regulations, purchase from multiple pharmacies, as well as the ease of availability over the internet, are a growing concern expressed by the pharmacy professionals. Similar concerns were voiced among pharmacists in the United Kingdom and Ireland^20^ as well as medical practitioners in South Africa.^21^

A level of caution appears to be exercised when patients request a product specifically by brand name. This was seen as the most frequent trigger experienced by the respondents for suspecting codeine misuse. According to Hamer et al.,^22^ most pharmacists will intervene on codeine overuse by asking the person to seek the doctor’s advice. Contrary to this, respondents of the present study indicated the use of referral system as the second least strategy applied. A study conducted in South Africa showed similar results with only 27% of the health professionals opting for referral in situations of suspected codeine misuse.^19^ Sub-optimal referral is linked to the lack of awareness of referral structures and appropriate addiction centres.^20^

A study conducted in Ireland, United Kingdom and South Africa^20^ showed that pharmacist intervention attempts centred on offering alternative first line pain management prior to supplying the codeine product or offering a product with similar packaging. In line with this, the present study found that after restricted supply (the most used intervention), the pharmacy professionals opt for substitution of codeine-containing medicines with non-opioid products. Hoppe et al.^12^ reported that 40% of the pharmacists counselled patients on the risks of taking excessive codeine-containing products. The result of the current study is in concordance with the reported literature. In addition to these most frequently used strategies, few participants reported using slow or gradual withdrawal of the codeine-containing products as an intervention.

### Limitations

The major limitation of the study was the response rate from both pharmacists and QPBPAs.

### Recommendations

Training and educating pharmacists and QPBPAs on identifying and assessing codeine misuse and dependence, on improved methods of communication with customers and on the provision of adequate patient information, is emphasised. Awareness campaigns on the availability of drug rehabilitation programmes and referral structures should be provided.

Further research is needed to establish patient knowledge and understanding of risks when taking medicines containing codeine.

Greater consideration towards enforcing current regulations pertaining to the dispensing of codeine-containing products and possible up-scheduling of these products be recommended to reduce the misuse.

## Conclusion

The results showed growing concern regarding the availability and accessibility of codeine-containing medicines within the community pharmacy sector and through other sources, such as the internet. Pharmacy professionals feel that the adverse health consequences of codeine misuse and dependence are not fully understood by customers, and the ineffectiveness of information provision by pharmacy personnel has been highlighted as a major concern. Currently, the referral structures are not utilised optimally by pharmacy professionals, who rather employ strategies such as refusal and substitution, which were not proved to be effective.

The continued availability of OTC codeine as a useful medicine is desirable. However, enforcement of strict regulations is needed to ensure appropriate use of codeine-containing medicines. The merits of up-scheduling codeine-containing medicines versus the need for the OTC availability of these medicines to the general public should be debated.
